# Poor Sleep quality and health-related quality of life impact in adolescents with and without chronic immunosuppressive conditions during COVID-19 quarantine

**DOI:** 10.6061/clinics/2021/e3501

**Published:** 2021-11-10

**Authors:** Alberto C. Helito, Livia Lindoso, Sofia M. Sieczkowska, Camilla Astley, Ligia B. Queiroz, Natalia Rose, Claudia Renata P. Santos, Thalis Bolzan, Rita María I.A. Peralta, Ruth R. Franco, Louise Cominato, Rosa Maria R. Pereira, Uenis Tannuri, Lucia Maria A. Campos, Benito Lourenço, Ricardo K. Toma, Karina Medeiros, Andréia Watanabe, Patricia Moreno Grangeiro, Sylvia C. Farhat, Caio B. Casella, Guilherme V. Polanczyk, Bruno Gualano, Clovis A. Silva, HC-FMUSP Adolescent COVID-19 Study Group, Adriana M. E. Sallum, Amanda Y. Iraha, Bianca P. Ihara, Bruna C. Mazzolani, Claudia A. Martinez, Claudia A. A. Strabelli, Claudia B. Fonseca, Dandara C. C. Lima, Debora N. D. Setoue, Deborah F. P. Roz, Fabiana I. Smaira, Hamilton Roschel, Helena T. Miyatani, Isabela G. Marques, Jane Oba, Juliana C. O. Ferreira, Juliana R. Simon, Katia Kozu, Ligia P. Saccani, Lorena V. M. Martiniano, Luana C. A. Miranda, Luiz E. V. Silva, Moisés F. Laurentino, Nadia E. Aikawa, Neusa K. Sakita, Nicolas Y. Tanigava, Paulo R. A. Pereira, Patrícia Palmeira, Simone S. Angelo, Sofia S. M. Lavorato, Tamires M. Bernardes, Tathiane C. Franco, Vivianne S. L. Viana, Vera P. M. F. R. Barros, Yingying Zheng

**Affiliations:** Hospital das Clinicas HCFMUSP, Faculdade de Medicina, Universidade de Sao Paulo, Sao Paulo, SP, BR.; Hospital das Clinicas HCFMUSP, Faculdade de Medicina, Universidade de Sao Paulo, SP, BR.

**Keywords:** COVID-19, Sleep, Adolescent, Chronic Condition, Health-Related Quality of Life

## Abstract

**OBJECTIVE:**

To assess the possible factors that influence sleep quality in adolescents with and without chronic immunosuppressive conditions quarantined during the coronavirus disease 2019 (COVID-19) pandemic.

**METHODS:**

This cross-sectional study included 305 adolescents with chronic immunocompromised conditions and 82 healthy adolescents. Online surveys were completed, which included questions on socio-demographic data and self-rated healthcare routine during COVID-19 quarantine and the following validated questionnaires: the Pittsburgh Sleep Quality Index (PSQI), Pediatric Quality of Life Inventory 4.0 (PedsQL4.0), and Pediatric Outcome Data Collection Instrument (PODCI).

**RESULTS:**

The median current age [14 (10-18) *vs*. 15 (10-18) years, *p*=0.847] and frequency of female sex (62% *vs*. 58%, *p*=0.571) were similar in adolescents with chronic conditions compared with healthy adolescents. The frequency of poor sleep quality was similar in both groups (38% *vs*. 48%, *p*=0.118). Logistic regression analysis, including both healthy adolescents and adolescents with chronic conditions (n=387), demonstrated that self-reported increase in screen time (odds ratio [OR] 3.0; 95% confidence interval [CI] 1.3-6.8; *p*=0.008) and intrafamilial violence report (OR 2.1; 95% CI 1.2-3.5; *p*=0.008) were independently associated with poor sleep quality in these adolescents. However, the PODCI global function score was associated with a lower OR for poor sleep quality (OR 0.97; 95% CI 0.94-0.99; *p*=0.001). Further logistic regression, including only adolescents with chronic conditions (n=305), demonstrated that self-reported increase in screen time (OR 3.1; 95% CI 1.4-6.8; *p*=0.006) and intrafamilial violence report (OR 2.0; 95% CI 1.2-3.4; *p*=0.011) remained independently associated with poor quality of sleep, whereas a lower PODCI global function score was associated with a lower OR for sleep quality (OR 0.96; 95% CI 0.94-0.98; *p*<0.001).

**CONCLUSION:**

Self-reported increases in screen time and intrafamilial violence report impacted sleep quality in both healthy adolescents and those with chronic conditions. Decreased health-related quality of life was observed in adolescents with poor sleep quality.

## INTRODUCTION

The new coronavirus disease (COVID-19) emerged in China at the end of 2019 and precipitated a global health crisis (1,2). Moreover, the policies adopted by governments to reduce the spread of this disease, such as social distancing, face mask use, stay-at-home orders, and closure of schools and leisure activities, resulted in abrupt changes in daily routines and lifestyles (2,3).

These changes might affect sleep patterns and physical and mental health and wellbeing (4). Indeed, during this pandemic, adolescents are experiencing more flexible awakening time, reduced exposure to sunlight, prolonged day naps, increased exposure to “blue light,” and disruption of melatonin production (5). Recent studies have reported that an increase in screen time (6), mental health disorders (5), and intrafamilial violence (7) may contribute to poor sleep quality in pediatric and adult populations during the COVID-19 pandemic.

Adolescents with chronic immunosuppressive conditions have complex diseases, requiring coordination of multiple specialties, humanized care, and different treatment approaches (8). The impact on physical health, sleep quality, and health-related quality of life (HRQL) parameters may affect these adolescents during this emergent crisis (9). However, to the best of our knowledge, the concomitant analysis of validated tools of sleep quality and HRQL was not systematically assessed in this vulnerable group quarantined during the COVID-19 pandemic.

Therefore, this study aimed to assess sleep quality parameters in adolescents with chronic immunosuppressive conditions and healthy adolescents during the COVID-19 quarantine period. We also evaluated the possible association of poor-quality sleep with demographic data, information about COVID-19 and HRQL parameters in adolescents with chronic immunosuppressive conditions.

## MATERIALS AND METHODS

From July to October 2020, a cross-sectional study using an online survey was performed with 704 adolescents aged 10-18 years during the COVID-19 pandemic in Brazil. Of these, 555 adolescents had chronic immunocompromised conditions and were followed at our tertiary and referral hospital (10,11); 250 adolescents were excluded because they did not answer the online survey (n=152) or because of incomplete survey data (n=98). Thus, 305 adolescents with chronic immunocompromised conditions comprised the study group.

The following chronic immunocompromised conditions were included in this study: rheumatic conditions (juvenile idiopathic arthritis, juvenile systemic lupus erythematosus, and juvenile dermatomyositis), kidney conditions (glomerulopathies, chronic kidney disease stages 4 and 5, and kidney transplant), and gastrointestinal and liver conditions (eosinophilic esophagitis, inflammatory bowel disease, celiac disease, autoimmune hepatitis, and liver transplant). The diagnosis of each disease was established according to specific classification criteria (12-20).

After approval of parents/legal guardians, 149 healthy adolescents were recruited by advertisement on radio, television, daily newspapers, Facebook, and Instagram. Of these healthy adolescents, 67 were excluded because of refusal to participate in the present study (n=23) and because of incomplete survey data (n=44). Therefore, 82 healthy adolescents were included in the control group. None of them reported a previous diagnosis of mental health disorders before the COVID-19 pandemic or other acute or chronic conditions.

The first contact with all participants was made by phone calls for a brief conversation. Psychological and psychiatric support was offered to adolescents with chronic immunosuppressed diseases and healthy adolescents, particularly those who reported any signs or symptoms of mental health issues.

To create and to distribute the survey, we used the Research Electronic Data Capture (REDCap^®^) application. The online survey was answered using cellphones, computers, or tablets. At least six emails or WhatsApp messages were forwarded to increase the response frequency. This study was approved by the Brazilian National Committee for Research Ethics (CONEP number: 4.081.961). All parents/legal guardians and adolescents provided informed consent and assent at the beginning of the online survey.

The online survey comprised four parts, predominantly based on information from the previous month. The estimated time for responses was approximately 45 min. The first part of the survey contained questions about socio-demographic data, school, healthcare routine, general information about COVID-19, impact of quarantine, and physical health during the COVID-19 pandemic ([Table t06] Appendix) for which the response formats were multiple choice, dichotomous (yes and no), or ordinal based on the visual analogue scale (VAS) (ranging from 0-10). Previous diagnoses of mental disorders before the COVID-19 pandemic were also systematically recorded.

The second part of the survey was composed of the Pittsburgh Sleep Quality Index (PSQI) (21), which comprises 19 questions regarding sleep quality and disorders. This instrument evaluates seven sleep categories: sleep latency, sleep duration, sleep efficiency, sleep disturbance, sleep medication use, overall sleep quality, and daytime dysfunction because of sleepiness. Each score varied from 0 to 3 for each element, and the PSQI total score ranged from 0 to 21. A total score >5 points was defined as poor sleep quality and ≤5 points as good sleep quality (18).

The third part of the survey was a self-report generic version of the Pediatric Quality of Life Inventory 4.0 (PedsQL) (22), which measured HRQL in four multidimensional scales: physical, emotional, social, and school functioning. The survey contains 23 items that are scored on a five-point scale. The sum of the two summary scores (physical health summary score and psychosocial health summary score) generates the total PedsQL score. The answers are given on a five-point scale ranging from 0 (“almost always”) to 100 (“never”), with higher scores indicating better HRQL.

The fourth part of the survey was a self-report version of the Pediatric Outcome Data Collection Instrument (PODCI) (23), a generic questionnaire with 83 questions developed to evaluate HRQL. It is divided into five subscales (upper extremity and physical functioning, transfer and basic mobility, sports and physical functioning, pain/comfort, and happiness) and a PODCI global function score, each ranging from 0 to 100. Lower scores indicated a lower HRQL.

### Statistical analyses

The sample size of 387 adolescents provided a power of 80% to find differences greater than 31% of poor sleep quality among adolescents with chronic immunocompromised conditions and healthy controls (GraphPad StatMate 1.01, GraphPad Software, Inc., CA, USA). Statistical analyses were performed using the Statistical Package for Social Sciences (SPSS) for Windows 24.0 (IBM Corp., Armonk, NY, USA). The results were described as numbers and frequencies for categorical variables and as median, minimum, and maximum values or means±standard deviations for continuous variables. The scores that had a non-normal distribution were compared using the Mann-Whitney test. Differences in categorical variables were evaluated using Fisher's exact test or Pearson’s chi-square test, as indicated. Spearman rank correlation coefficient was used for analyzing the correlation between the total PSQI score and PedsQL, PODCI, and VAS (physical activity [PA] scale, disease activity/complication, fear of immunosuppressive use, and fear of COVID-19). Logistic regression analysis models (forward stepwise) were performed using poor sleep quality as a dependent variable and variables that presented a statistical significance level of *p*<0.2 in the univariate analyses as independent variables, in adolescents with chronic immunosuppressive conditions and healthy controls. Statistical significance was set at *p*<0.05.

## RESULTS

The median current age [14 (10-18) *vs*. 15 (10-18) years, *p*=0.847] and frequency of female sex (62% *vs*. 58%, *p*=0.571) were similar in adolescents with chronic conditions compared with healthy adolescents (Table 1). Adolescents with the following diagnoses of chronic immunocompromising conditions were included: juvenile idiopathic arthritis, n=81 (27%); juvenile systemic lupus erythematosus, n=41 (13%); juvenile dermatomyositis, n=23 (7%); kidney conditions, n=26 (9%); eosinophilic esophagitis, n=21 (7%); inflammatory bowel disease, n=35 (12%); celiac disease, n=11 (4%); autoimmune hepatitis, n=23 (7%); and liver transplant, n=44 (14%).

Demographic data and the PSQI, PedsQL, and PODCI scores reported by adolescents with chronic immunosuppressive conditions *versus* those reported by healthy adolescents during the COVID-19 quarantine are shown in Table 1. The frequencies of poor sleep quality were similar in both groups (38% *vs.* 48%, *p*=0.118), whereas the means of sleep latency (1.11±1.02 *vs.* 1.37±1.05, *p*=0.043) and day dysfunction because of sleepiness (0.71±0.78 *vs.* 1.01±0.87, *p*=0.003) according to PSQI score were significantly lower in adolescents with chronic immunosuppressive conditions than in healthy controls. Adolescents with chronic conditions had a higher median happiness scale by the PODCI than healthy controls [85 (5-100) *vs*. 75 (0-100), *p*=0.025]. Additional evaluations of poor sleep quality, the PSQI total score, the PedsQL total scale score and its subscales, and the PODCI global function score and its subscales were similar in both groups (all *p*>0.05, Table 1).

Univariate analysis, including both adolescents with chronic conditions and healthy controls (n=387), showed that self-reported increases in screen time during the pandemic [odds ratio (OR) 2.6; 95% confidence interval (CI) 1.3-5.5; *p*=0.01], intrafamilial violence report (OR 2.0; 95% CI 1.2-3.3; *p*=0.01), and female sex (OR 1.7; 95% CI 1.1-2.5; *p*=0.02) had increased odds of poor sleep quality in these adolescents. However, the PODCI global function score was associated with lower odds of poor sleep quality (OR, 0.96; 95% CI, 0.94-0.99; *p*=0.001).

Further comparisons between adolescents with chronic immunosuppressive conditions and poor sleep quality (total PSQI score >5) compared with those with good sleep quality (total PSQI score ≤5) during the COVID-19 quarantine showed that the frequency of adolescents in public school was significantly higher in the former group (79% *vs*. 68%, *p*=0.036). Moreover, adolescents with chronic immunosuppressive conditions and poor sleep quality reported higher frequencies of increase in screen time (92% *vs*. 83%, *p*=0.023), intrafamilial violence (28% *vs*. 15%, *p*=0.009), and higher median fear of immunosuppressive medication use [5.0 (0-10) *vs*. 3.8 (0-10), *p*=0.034) during the COVID-19 pandemic (Table 2).

The median PedsQL, PODCI, and their subscales were significantly lower in adolescents with chronic immunosuppressive conditions and poor sleep quality than in those with good sleep quality during the COVID-19 quarantine (*p*<0.05), except for transfer and basic mobility of the PODCI scores (*p*=0.055, Table 3).

Further univariate analysis, including only adolescents with chronic immunosuppressive conditions (n=305), revealed that self-reported increases in screen time during the pandemic [OR 2.4; 95% CI 1.1-5.3; *p*=0.03], intrafamilial violence (OR 2.1; 95% CI 1.2-3.7; *p*=0.01), public school attendance (OR 1.8; 95% CI 1.1-3.1; *p*=0.04), and fear of immunosuppression use (OR 1.0; 95% CI 1.01-1.15; *p*=0.02) had increased odds of poor sleep quality in these adolescents. However, the PODCI global function score was associated with a lower odds of poor sleep quality (OR, 0.97; 95% CI, 0.95-0.99; *p*=0.004).

Adolescents who presented with signs or symptoms of mental health issues were systematically assessed by pediatric psychologists. Eighteen adolescents with chronic conditions and healthy controls had private online appointments with psychologists, including eight of whom reported intrafamilial violence in the online survey. None of the eight adolescents with chronic conditions reported any kind of violence during appointments with pediatric psychologists.

Spearman correlations between the PSQI total score and independent variables are shown in Table 4. The final models of logistic regression analyses to evaluate independent factors associated with poor sleep quality are included in Table 5.

## DISCUSSION

Our study was the first to evaluate adolescents with chronic immunosuppressive conditions during the COVID-19 pandemic and the temporal relationship between various dimensions of sleep quality using PSQI and HRQL instruments.

One strength of this study was the use of self-report validated instruments with high reliability to measure sleep quality and HRQL. In addition, the current report evaluated a vulnerable and high-risk subgroup of adolescents who had more severe restrictions during the COVID-19 quarantine because of their chronic immunosuppressed conditions. Furthermore, the inclusion of a healthy control group adjusted for age and sex allowed a more precise analysis of the influence of chronic immunosuppressive conditions on sleep quality, as demographic features may impact sleep problems differently (5,6).

According to the PSQI results, approximately 40% of adolescents with chronic immunocompromised conditions and healthy adolescents had poor sleep quality during the COVID-19 pandemic. Healthy adolescents demonstrated higher sleep latency and daytime sleepiness than chronic immunosuppressed patients.

The COVID-19 pandemic caused by the new severe acute respiratory syndrome coronavirus 2 (SARS-CoV-2) has led to numerous health problems and decreased HRQL parameters among the general public worldwide (5,6,24,25). Our study demonstrated that the COVID-19 pandemic equally impacted HRQL in adolescents with chronic conditions and healthy adolescents. However, adolescents with chronic immunosuppressive conditions reported higher happiness scores by the PODCI than their healthy peers. Adolescents with chronic illnesses may have more coping mechanisms, since they learn how to deal with suffering and pain from a young age (26). Indeed, a nationwide German study found that children and adolescents felt considerably burdened because of quarantine measures, and experienced lower HRQL parameters, poor sleep quality, and mental health issues (27). Further analysis revealed that adolescents with chronic conditions and poor sleep quality had a lower total PedsQL, demonstrating the influence of sleep in general HRQL. Therefore, it is important to educate adolescents and to reinforce sleep hygiene measures during medical appointments.

We confirmed previous studies regarding the influence of screen time on sleep quality in healthy adolescents and those with chronic immunosuppressive conditions. Zhou et al. found that healthy adolescents and young adults who had increased mobile phone use, playing online games, and online shopping suffered from decreased sleep hygiene and quality and prolonged sleep latency (5). Additionally, a study from Chile reported that an increase in screen time was associated with a decline in sleep quality in children (6). Moreover, Nagata et al. showed that an increase in screen time may decrease physical activity in adolescents with obesity (28).

Of note, to the best of our knowledge, our study is the first to find an association between poor sleep quality and self-reported increase in screen time in adolescents with various chronic immunosuppressed conditions. This result may be related to the fact that more time expended in front of screens could lead adolescents to have less time to sleep (29). In addition, adolescents with chronic conditions and poor sleep quality attended predominantly public schools, and the absence of school activities during this cross-sectional study may have contributed to insomnia and sleep issues in this population.

During the COVID-19 pandemic period, studies have shown that increased sedentary behavior can influence sleep patterns in pediatric populations. Our study found an inverted association between the PSQI total score and increased physical activity per week using the VAS. Similarly, López-Bueno et al. demonstrated that a decrease in health-related behaviors leads to a reduction in weekly minutes of physical activity (30). Moreover, a study conducted in Verona revealed that physical activity was reduced by 2.5h per week during quarantine (31). Physical activity is crucial to healthy living and noticeably decreases the risk of systemic inflammation (32). Consequently, physical activity has a potential role in immune functions that are helpful in reducing the risk of infectious diseases from the perspective of the COVID-19 outbreak. Therefore, it is important to implement policies that encourage the practice of indoor exercise.

The COVID-19 pandemic has collateral effects ranging beyond those of viral infections. Importantly, intrafamilial violence reported by healthy adolescents and those with chronic immunosuppressive conditions impacted their quality of sleep. In fact, violence against adolescents is a serious public health, human rights, and social issue, with numerous traumatic consequences. However, it has rarely been reported in adolescents with multiple complex conditions. A recent systematic review studied the most important negative effects of school closings and social distancing during the COVID-19 pandemic, and suspected domestic violence was observed with an increase in domestic accidents and head trauma (33). Further qualitative studies, including ethical considerations for adolescents with private structured interviews, will be performed for this vulnerable population to clarify these findings and to assess the spectrum of violence, including physical/emotional aggression, negligence, and sexual abuse. If it is necessary for their own protection, adolescents experiencing violence will be promptly referred to the responsible legal sectors, such as the Protective Council and Child and Youth Court.

This study has some limitations. Our data came from only one tertiary center, which could have led to a selection bias. The cross-sectional design limited our ability to ascertain the causal relationship between the COVID-19 pandemic and changes in HRQL and sleep quality. During the COVID-19 pandemic, adolescents were overloaded with online activity, which could have affected the ratio of responses to the questionnaires. We only have data from one period of the COVID-19 pandemic, making it impossible to objectively compare with previous baseline situations. We did not assess generic or specific mental health tools, especially validated instruments concerning anxiety, depression, and coping/resilience. The online survey is another limitation of this study, because of the self-report nature of the questionnaires, at only a single instance. There is a risk of recall bias. We also analyzed only one question regarding self-reported increase in screen time during the pandemic and did not assess the total period of screen time, as well as television time, smartphone use, and computer use.

In conclusion, self-reported increases in screen time and intrafamilial violence report impacted sleep quality in both healthy adolescents and those with chronic conditions. Decreased HRQL was observed in adolescents with poor sleep quality.

### HC-FMUSP Adolescent COVID-19 Study Group:

Adriana M. E. Sallum, Amanda Y. Iraha, Bianca P. Ihara, Bruna C. Mazzolani, Claudia A. Martinez, Claudia A. A. Strabelli, Claudia B. Fonseca, Dandara C. C. Lima, Debora N. D. Setoue, Deborah F. P. Roz, Fabiana I. Smaira, Hamilton Roschel, Helena T. Miyatani, Isabela G. Marques, Jane Oba, Juliana C. O. Ferreira, Juliana R. Simon, Katia Kozu, Ligia P. Saccani, Lorena V. M. Martiniano, Luana C. A. Miranda, Luiz E. V. Silva, Moisés F. Laurentino, Nadia E. Aikawa, Neusa K. Sakita, Nicolas Y. Tanigava, Paulo R. A. Pereira, Patrícia Palmeira, Simone S. Angelo, Sofia S. M. Lavorato, Tamires M. Bernardes, Tathiane C. Franco, Vivianne S. L. Viana, Vera P. M. F. R. Barros, Yingying Zheng. All investigators are from Hospital das Clinicas HCFMUSP, Faculdade de Medicina, Universidade de Sao Paulo, SP, BR.

## AUTHOR CONTRIBUTIONS

All of the authors approved the final draft of the manuscript, approved its submission to the journal, and can take responsibility for it in its entirety. All authors contributed substantially to the conception and design of the study and in the analysis and interpretation of data. All authors revised the report critically and approved the final version of the manuscript.

## Figures and Tables

**Table 1:** Demographic data and the Pittsburgh Sleep Quality Index (PSQI), Pediatric Quality of Life Inventory 4.0 (PedsQL), and Pediatric Outcome Data Collection Instrument (PODCI) scores reported by adolescents with preexisting chronic conditions *versus* those reported by healthy adolescents during the coronavirus disease 2019 (COVID-19) quarantine.

Domains	Adolescents with preexisting chronic conditions (n=305)	Healthy Adolescents (n=82)	*p*
Demographic data			
Current age	14 (10-18)	15 (10-18)	0.847
Female sex	189 (62)	48 (58)	0.571
PSQI score			
PSQI total score (0-21)	5.16±3.34	5.87±3.49	0.092
Poor sleep quality, total score >5	116 (38)	39 (48)	0.118
Sleep latency (0-3)	1.11±1.02	1.37±1.05	0.043
Sleep duration (0-3)	0.17±0.60	0.27±0.60	0.202
Sleep efficiency (0-3)	0.52±0.94	0.68±0.92	0.155
Sleep disturbances (0-3)	1.08±0.50	1.15±0.57	0.272
Sleep medication use (0-3)	0.70±1.20	0.46±1.00	0.102
Overall sleep quality (0-3)	0.87±0.80	0.93±0.81	0.582
Day dysfunction because of sleepiness (0-3)	0.71±0.78	1.01±0.87	0.003
PedsQL score			
Total scale score (0-100)	73 (21-100)	70 (41-100)	0.415
Physical health summary score (0-100)	78 (6-100)	81 (38-100)	0.385
Psychosocial health summary (0-100)	70 (13-100)	70 (25-100)	0.786
Emotional functioning (0-100)	60 (0-100)	60 (10-100)	0.411
Social functioning (0-100)	85 (10-100)	85 (20-100)	0.894
School functioning (0-100)	65 (10-100)	65 (0-100)	0.157
PODCI score			
Global function score (0-100)	93 (32-100)	94 (64-100)	
Upper extremity and physical functioning (0-100)	100 (46-100)	100 (75-100)	0.627
Transfer and basic mobility (0-100)	100 (5-100)	100 (50-100)	0.499
Sports and physical functioning (0-100)	88 (3-100)	88 (33-100)	0.115
Pain/comfort (0-100)	93 (8-100)	92.5 (44-100)	0.621
Happiness (0-100)	85 (5-100)	75 (0-100)	0.025

Results are presented as medians (minimum-maximum values), means±standard deviations, and n (%).

**Table 2:** Demographic data and information on the coronavirus disease 2019 (COVID-19) pandemic reported by adolescents with preexisting chronic conditions and poor sleep quality (total PSQI score >5) *versus* those reported by adolescents with preexisting chronic conditions and good sleep quality (total PSQI score ≤5).

Variables	Adolescents with chronic conditions and poor sleep quality (n=116)	Adolescents with chronic conditions and good sleep quality (n=189)	*p*
Socio-demographic			
Current age	15 (10-18)	14 (10-18)	0.369
Female sex	78 (67)	111 (59)	0.137
Caucasians	58 (50)	98 (52)	0.753
Number of households in the residence			
≤3	37 (32)	63 (33)	0.795
>3	79 (68)	126 (67)	
School			
Level of schooling			0.546
Elementary school	65 (56)	115 (61)	
Middle school	43 (37)	56 (30)	
High school	5 (4)	12 (6)	
Not studying	3 (3)	6 (3)	
Online learning during the COVID-19 pandemic	104 (90)	156 (82)	0.098
Public school adolescents	92 (79)	129 (68)	0.036
Healthcare routine during the pandemic			
Medical appointment during the pandemic			0.768
Discontinued	38 (36)	70 (39)	
Decreased	41 (38)	70 (39)	
Unchanged	28 (26)	41 (22)	
Forgetting to take your medication			0.139
Without forgetting	64 (63)	118 (71)	
1-2 days	31 (30)	45 (27)	
≥3 days	7 (7)	4 (2)	
General information of the COVID-19 pandemic			
Reliable COVID-19 information	84 (72)	142 (75)	0.854
Compliance to “stay-home” policy	111 (96)	183 (97)	0.606
Impact of COVID-19 quarantine			
Households with COVID-19	22 (19)	24 (13)	0.138
Life routine changed after the “physical distancing” policy	109 (94)	176 (93)	0.773
Housework			
No housework activities	25 (22)	44 (23)	0.726
Do housework activities	91(78)	145 (77)	
Sleep duration			
≤8 h/day	57 (49)	48 (25)	<0.001
>8 h/day	59 (51)	141 (75)	
Sleep after midnight	85 (73)	103 (55)	0.001
Self-reported increase in screen time during the pandemic	107 (92)	157 (83)	0.023
Household worked out of home	104 (90)	160 (85)	0.214
Intrafamilial violence report during the pandemic	32 (28)	29 (15)	0.009
VAS (0-10)			
Fear of underlying disease activity/complication	7.2 (0-10)	5.1 (0-10)	0.053
Fear of immunosuppression use	5.0 (0-10)	3.8 (0-10)	0.034
Fear of COVID-19	6.5 (0-10)	6.5 (0-10)	0.654
Physical activity per week	2.1 (0-10)	2.7 (0-10)	0.104
Sleep quality	6.6 (0-10)	9.6 (0-10)	<0.001
Preexisting chronic conditions			
Gastrointestinal and liver conditions	59 (51)	75 (40)	0.160
Rheumatologic conditions	48 (41)	97 (51)	
Kidney conditions	9 (8)	17 (9)	
Previous psychiatric disorder	12 (48)	13 (52)	0.284

Results are presented as n (%), medians (minimum-maximum values), means±standard deviations, NA: not applicable to assess Pearson’s chi-square test, VAS: visual analogue scale in the last month (scale 0-10), and PSQI: Pittsburgh Sleep Quality Index.

**Table 3:** Pediatric Quality of Life Inventory 4.0 (PedsQL) and Pediatric Outcome Data Collection Instrument (PODCI) scores reported by adolescents with preexisting chronic conditions and poor sleep quality (total PSQI score >5) *versus* those reported by adolescents with preexisting chronic conditions and good sleep quality (total PSQI score ≤5) during quarantine because of the coronavirus disease 2019 (COVID-19) pandemic.

Domains	Adolescents with chronic conditions and poor sleep quality (n=116)	Adolescents with chronic conditions and good sleep quality (n=188)	*p*
PedsQL score			
Total scale score (0-100)	67 (21-93)	77 (32-100)	<0.001
Physical health summary score (0-100)	72 (22-100)	81 (6-100)	<0.001
Psychosocial health summary (0-100)	63 (13-93)	73 (35-100)	<0.001
Emotional functioning (0-100)	55 (0-95)	70 (10-100)	<0.001
Social functioning (0-100)	82.5 (10-100)	90 (35-100)	0.003
School functioning (0-100)	60 (10-100)	67.5 (15-100)	<0.001
PODCI score	(n=113)	(n=177)	
Global function score (0-100)	90 (40-100)	95 (32-100)	<0.001
Upper extremity and physical functioning (0-100)	100 (46-100)	100 (63-100)	0.039
Transfer and basic mobility (0-100)	100 (49-100)	100 (5-100)	0.055
Sports and physical functioning (0-100)	83 (14-100)	89 (3-100)	0.020
Pain/comfort (0-100)	78 (8-100)	100 (19-100)	<0.001
Happiness (0-100)	80 (5-100)	90 (15-100)	<0.001

Results are presented as medians (minimum-maximum values) and Pittsburgh Sleep Quality Index (PSQI) scores.

**Table 4:** Correlations between the PSQI total score and PedsQL total scale and independent variables.

Variables	r Spearman	*p*
PSQI total score and psychosocial health summary by PedsQL	-0.459	<0.001
PSQI total score and physical health summary score by PedsQL	-0.308	<0.001
PSQI total score and total scale score by PedsQL score	-0.413	<0.001
PSQI total score and happiness by PODCI score	-0.328	<0.001
PSQI total score and global function score by PODCI score	-0.255	<0.001
PSQI total score and increase PA per week by VAS	-0.149	0.009
PSQI total score and fear of immunosuppressive use by VAS	+0.137	0.019
PSQI total score and current age	+0.124	0.031
PSQI total score and fear of underlying disease activity or complication by VAS	+0.115	0.049

r Spearman - Spearman rank correlation coefficients, PSQI: Pittsburgh Sleep Quality Index, PedsQL: Pediatric Quality of Life Inventory 4.0, PODCI: Pediatric Outcome Data Collection Instrument, VAS: visual analogue scale, + positive, - negative

**Table 5:** Final models of logistic regression analyses to evaluate independent variables associated with poor sleep quality.

	Both adolescents with preexisting chronic conditions and healthy controls Dependent variable - poor sleep quality (n=387)		
Independent variables	Odds ratio	95% CI	*p*
Self-reported increase in screen time during the pandemic	3.0	1.3-6.8	0.008
Intrafamilial violence report during the pandemic	2.1	1.2-3.5	0.008
PODCI global function score	0.97	0.94-0.99	0.001

	Solely adolescents with preexisting chronic conditions Dependent variable - poor sleep quality (n=305)		
Independent variables	Odds ratio	95% CI	*p*
Self-reported increase in screen time during the pandemic	3.1	1.4-6.8	0.006
Intrafamilial violence report during the pandemic	2.0	1.2-3.4	0.011
PODCI global function score	0.96	0.94-0.98	<0.001

CI: confidence interval.

## APPENDIX

### Semi-structured questionnaire

Hello, [name of patient]!

We would like you to fill this questionnaire today.

Please take note of any doubts while filling the questionnaire or concerning any of the answers.

We’ll get in touch as soon as possible.

**Table t06:**

Age in years	_____________________________________ (use only numbers)
Gender	○ Woman
	○ Man
	○ Other gender identification
Would you like to tell us more about your gender?	_____________________________________
Sex	○ Female
	○ Male
	○ Other
	○ I’m not comfortable to answer
Describe any other	_____________________________________
Skin color	○ White ○ Black ○ Yellow
	○ Indian ○ Other
Were you attending school this year, before the new coronavirus pandemic?	○ No ○ Yes
What is your school year?	○ Fundamental school - 1st grade
	○ Fundamental school - 2nd grade
	○ Fundamental school - 3rd grade
	○ Fundamental school - 4th grade
	○ Fundamental school - 5th grade
	○ Fundamental school - 6th grade
	○ Fundamental school - 7th grade
	○ Fundamental school - 8th grade
	○ Fundamental school - 9th grade
	○ High school - 1st grade
	○ High school - 2nd grade
	○ High school - 3rd grade
	○ Incomplete university education
	○ Complete university education
	○ I am not studying
Do you study at	○ Public school ○ Private school
Have you been followed-up by our team for any disease?	○ No ○ Yes
What disease(s) or condition(s) do you treat?	_____________________________________
Do you use any routine medications?	○ No ○ Yes
Tell us what routine medications you use.	[name of medication, dose and frequency (daily, weekly or monthyl)]
Before the coronavirus pandemic, how often did you go to the hospital for appointments or treatment?	○ More than once a month
	○ Once a month
	○ Once every 2 months
	○ Once every 3 months
	○ Once every 4 months or less often
How about after the pandemic started? How often are you going to the hospital?	○ I’m not going ○ I’m going as before ○ I’m going less often
In a whole week, how often do you forget to take your medications?	○ Once a week
	○ Twice a week
	○ Three times a week
	○ Four times a week or more often
	○ I never forget
Did you take the Flu vaccine?	○ No ○ Yes
What are your sources of information about the coronavirus and social distancing?	○ Television ○ Radio
	○ Internet ○ Family members
	○ Friends ○ WhatsApp
	○ Social media such as Facebook, Instagram, Twitter, etc.
	○ Healthcare professionals such as doctors, nurses, social workers, etc.
Do you believe having enough and reliable information about the new coronavirus and COVID-19?	○ No
	○ Yes
	○ I don’t know
If not, tell us why:	_____________________________________
Write your doubts concerning the new coronavirus and COVID-19.	_____________________________________
Do you agree with the “STAY HOME” policy, made by health authorities and the government?	○ No ○ Yes
Is or was there someone in your home who was infected by the coronavirus?	○ No ○ Yes
How is quarantine being made in your home?	_____________________________________
The infected person/persons required hospital admission?	○ No ○ Yes
Where were they admitted?	_____________________________________
Was it easy to find a hospital able to admit them?	○ No ○ Yes
How did they evolve?	○ They got well
	○ They are still recovering
	○ They did not get well
Did your life routine change after physical distancing measures adopted by our country?	○ No ○ Yes

**Table:** About your daily routine, how much time are you dedicating for the following activities?.

Remote school activities?	○ I don’t have remote school activities
	○ Less than 3 hours a day
	○ More than 3 hours a day
Domestic tasks?	○ I don’t do domestic tasks
	○ Less than 1 hours a day
	○ 2 or 3 hours a day
	○ more than 3 hours a day
Taking care of elders?	○ I don’t take care of the elder
	○ Less than 1 hours a day
	○ Between 1 and 2 hours a day
	○ Between 2 and 3 hours a day
	○ more than 3 hours a day
How much physical activities do you do per week in a scale from 0 to 10 (0=without any physical activity to 10=vigorous physical activity daily)?	
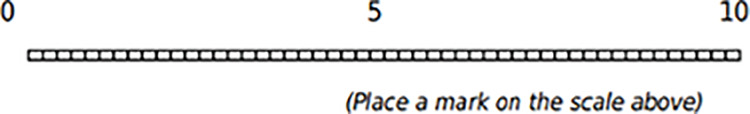
How is your sleep?	○ Sleep more than 8 hours a day
	○ Sleep less than 8 hours a day
Do you usually sleep after midnight?	○ No ○ Yes
How is your sleep in a scale from 0 to 10 (0=without difficulties to 10=severe insomnia)?	
Are you experiencing trouble to sleep?	○ No ○ Yes
Use of electronic devices and screen time for accessing social media, WhatsApp, music platforms, electronic games, watching series, movies and videos.	○ Less than 3 hours a day
	○ Between 3 and 4 hours a day
	○ Between 4and 5 hours a day
	○ Between 5 and 6 hours a day
	○ More than 6 hours a day
Do you think your screen time increased after social distancing?	○ No ○ Yes
Concerning the use of alcohol during physical distancing:	○ I don’t drink alcohol
	○ I’m drinking more
	○ I’m drinking less
	○ I’m drinking the same amount
How many rooms are there in your home?	○ 1 ○ 2 ○ 3 ○ 4
	○ 5 ○ 6 ○ 7 ○ 8
	○ 9 ○ 10
How many people live in your home?	○ 1 ○ 2 ○ 3 ○ 4 ○ 5 ○ 6 ○ 7 ○ 8
	○ 9 ○ 10 ○ 11 ○ 12 ○ 13 ○ 14 ○ 15
Your family’s financial situation (money earning) after social distancing is:	○ Worse
	○ Better
	○ The same
Is anyone who lives with you going out to work?	○ No ○ Yes
Is anyone who lives with you smoking?	○ No ○ Yes
Who is the smoker?	○ Mother
	○ Father
	○ Other
Define:	_____________________________________
Where is he/she smoking?	○ Bedroom ○ Living room ○ Bathroom
	○ Laundry ○ Balcony ○ Street
Is there factories or industry in the two blocks near your home, during quarantine?	○ No ○ Yes
Are there gas stations in the two blocks near your home, during quarantine?	○ No ○ Yes
Concerning the fear of getting infected by the coronavirus, how are you feeling now in a scale from 0 to 10 (0=no fear to 10=with extreme fear)?	
How is your fear of getting worse of your disease or condition during the coronavirus pandemic in a scale from 0 to 10 (0=no fear to 10=with extreme fear)?	
How is your fear related to immunosuppressive medications use during the coronavirus pandemic in a scale from 0 to 10 (0=no fear to 10=with extreme fear)?	
Did you notice any increase in conflicts or violence in your home or neighborhood after the beginning of physical distancing?	○ No ○ Yes
Would you like to talk to someone about that?	○ No ○ Yes
Describe those conflicts and violence.	_____________________________________
What do you miss the most during the distancing?	_____________________________________
What changed about your friendships?	_____________________________________
What changed about your dating/making out habits?	_____________________________________
How is your family treating you in this moment?	_____________________________________
Write freely to tell us about what you have been feeling during this period of social distancing.	_____________________________________
How did you like this questionnaire?	_____________________________________
